# Continuous deep sedation at the end of life: a qualitative interview-study among health care providers on an evolving practice

**DOI:** 10.1186/s12904-023-01289-z

**Published:** 2023-10-26

**Authors:** Madelon T Heijltjes, Ghislaine JMW van Thiel, Judith AC Rietjens, Agnes van der Heide, Geeske Hendriksen, Johannes JM van Delden

**Affiliations:** 1 Department of Bioethics and Health Humanities, Julius Center for Health Sciences and Primary Care, UMC Utrecht, PO Box 85500, Utrecht, 3508 GA The Netherlands; 2 Department of Public Health, Erasmus Medical Center, Rotterdam, the Netherlands

**Keywords:** Continuous sedation, Deep sedation, Palliative sedation, Terminal sedation, Qualitative research, Health care professional, End of life

## Abstract

**Background:**

Continuous deep sedation (CDS) can be used for patients at the end of life who suffer intolerably from severe symptoms that cannot be relieved otherwise. In the Netherlands, the use of CDS is guided by an national guideline since 2005. The percentage of patients for whom CDS is used increased from 8% of all patients who died in 2005 to 18% in 2015. The aim of this study is to explore potential causes of the rise in the use of CDS in the Netherlands according to health care providers who have been participating in this practice.

**Methods:**

Semi-structured interviews were conducted and thematically analysed. Participants were Dutch health care providers (HCPs), working at patients’ homes, hospices, elderly care facilities and in hospitals and experienced in providing CDS, who were recruited via purposeful sampling.

**Results:**

41 Health care providers participated in an interview. For these HCPs the reason to start CDS is often a combination of symptoms resulting in a refractory state. HCPs indicated that symptoms of non-physical origin are increasingly important in the decision to start CDS. Most HCPs felt that suffering at the end of life is less tolerated by patients, their relatives, and sometimes by HCPs; they report more requests to relieve suffering by using CDS. Some HCPs in our study have experienced increasing pressure to perform CDS. Some HCPs stated that they more often used intermittent sedation, sometimes resulting in CDS.

**Conclusions:**

This study provides insight into how participating HCPs perceive that their practice of CDS changed over time. The combination of a broader interpretation of refractory suffering by HCPs and a decreased tolerance of suffering at the end of life by patients, their relatives and HCPs, may have led to a lower threshold to start CDS.

**Trial registration:**

The Research Ethics Committee of University Medical Center Utrecht assessed that the study was exempt from ethical review according to Dutch law (Protocol number 19–435/C).

**Supplementary Information:**

The online version contains supplementary material available at 10.1186/s12904-023-01289-z.

## Introduction

Patients at the end of life may suffer intolerably from severe symptoms that cannot be relieved by conventional treatment options [1, 2]. Continuous deep sedation (CDS) can be used to relieve such suffering. With CDS, the patient is deeply sedated until the end of life. This form of sedation is often distinguished from other types of palliative sedation, such as intermittent or superficial sedation [3–6]. The fact that CDS implies that patients lose their ability to communicate and the possibility that CDS could hasten a patient’s death have been sources of debate about the appropriate use of this intervention for years [7–9].

To guide a responsible practice, the Royal Dutch Medical Association issued a guideline on palliative sedation in 2005, with updated versions in 2009 and 2022 [10–12]. In this guideline, different forms of palliative sedation are addressed, including CDS. Core elements of the guideline are presented in Table 1. The guideline provides information for health care providers (HCPs) about various types of palliative sedation, indications and contraindications, the appropriate medication, and practical procedures. Core elements of the guideline remained unchanged in the 2009 and 2022 versions.

In the Netherlands, the use of CDS increased from 8% of all patients who died in 2005 to 18% of all patients who died in 2015. A systematic review suggests that the use of CDS increases on an international level, and that a broadening of indications to start CDS is visible, from only physical symptoms to also symptoms of non-physical origin [13]. An international questionnaire study among physicians showed that a substantial proportion of physicians considered the use of CDS an acceptable practice to relieve symptoms of physical and non-physical origin [14]. Little is known about why the use of CDS increased in the Netherlands over the years. The aim of our study is to explore potential causes of the rise in the use of CDS in the Netherlands according to health care providers who have been participating in this practice.

## Methods

### Design

We performed a qualitative interview study among Dutch health care providers (HCPs) experienced in providing CDS. The interviews were conducted by the use of a topic-list. The topic list was designed for this study and was refined after three pilot-interviews (supplementary file 1).To gain insight in current practice, respondents were asked to reflect on their most recent case of CDS. In addition, respondents were asked to reflect on their general views on and practice of CDS, and if these had changed over the years. We report the study according to the COnsolidated criteria for REporting Qualitative research (COREQ) [15].

#### Sample of respondents

We recruited respondents via purposeful sampling, through key persons in health care organizations, and via snowballing. Via purposeful sampling we invited health care providers in our network to participate in an interview. To acquire a broad range of perspectives, we invited general practitioners, nursing home physicians, medical specialists, physician assistants, nurses, and spiritual carers involved in the care for terminally ill patients. Inclusion criteria were that these health care providers had actual experience with CDS, and that they had several years of work experience in their field so that they could reflect on changes in their use of CDS. We also recruited respondents via key persons in health care organizations. These key persons were HCPs who fulfilled a coordinating role in their organization. They worked at patients’ homes, hospices, elderly care facilities and in hospitals. Inclusion criteria were that they had to be HCPs experienced with providing CDS.

### Data collection

The interviews were conducted face to face and from March 2020 onwards also online due to the Covid-19 pandemic. The interviews were conducted by MH, who completed training in qualitative research. MH is a female physician, at the time working as a fulltime PhD student. MH contacted respondents prior the interview by telephone or by email, to clarify the research topic. Researcher reflexivity was enhanced by debriefing the interviews in meetings of the authors. The interviews were recorded, transcribed verbatim and anonymized. Background details of the respondents were obtained from an additional questionnaire. The Research Ethics Committee of University Medical Center Utrecht assessed that the study was exempt from ethical review according to Dutch law (Protocol number 19–435/C). Respondents provided written informed consent prior to participating in an interview.

### Data analysis

We performed a thematic analysis to gain insight in different perspectives of respondents and to highlight similarities and differences [16]. The 2009 guideline of the RDMA on palliative sedation served as the conceptual framework for this study Table 1). To promote rigor, credibility and trustworthiness, several transcripts were closely (re)read by the entire team during all steps. The analysis consisted of four steps and was partly deductive, as the topic-list was based on relevant themes in the literature, and partly inductive, as during the analysis new themes and subthemes arose. First, interviews were read and reread to get familiar with the data. Second, two researchers (MH and LN) independently coded the transcripts by assigning descriptive codes to interview fragments, using Nvivo 12. In addition, GvT coded five interviews. Third, MH collated the codes and merged them into themes. These themes were discussed and refined through critical dialogue by the research team. The code tree was evaluated regularly during this second and third step. Fourth, key themes were identified and discussed in weekly meetings of MH and GvT, and in monthly meetings of all team members. Data saturation on a conceptual level was achieved, as in the last interviews with HCPs from different groups no new concepts or perspectives came up anymore.

**Table 1:** Core elements of the 2009 version of the RDMA guideline on the use of CDS^A^

• Continuous sedation is always administered in the final stage of life. The patients concerned are dying and experiencing unbearable suffering • Medical indications are present when one or more intractable or ‘refractory’ symptoms are causing the patient unbearable suffering. A symptom is considered to be refractory if none of the conventional modes of treatment is effective or fast acting enough, and/or if these modes of treatment are accompanied by unacceptable side-effects • A precondition for the use of continuous sedation is the expectation that death will ensue in the reasonably near future − that is, within one to two weeks. Next to physical suffering, existential suffering can also play a role in determining if suffering is unbearable and refractory. However, existential suffering alone cannot be an indication to start continuous sedation. When patients suffer from existential problems, it is recommended to consult an expert in psychosocial and spiritual care • Palliative sedation is a medical response to a serious medical problem. A patient cannot opt for continuous sedation unless the indications and preconditions for this option are fulfilled. Only if the indications are present, in the physician’s opinion, and the preconditions have been met does continuous sedation become a right that the patient may choose to exercise. • The general rule is that palliative sedation should not be initiated without the consent either of the patient himself or, if he is decisionally incompetent, his representative. The patient’s condition may make it necessary to administer acute sedation. This means sedating a patient in a situation in which a complication (frequently one that is life-threatening) suddenly occurs that causes unbearable suffering. In that case, the physician may decide that acute sedation is the only sound option for alleviating the patient’s suffering at the point in time. • Where a physician has doubts regarding his own expertise or has difficulty balancing the different considerations involved in deciding whether to start CDS, it is standard professional practice to consult the appropriate expert in good time • Midazolam is the drug of choice, the use of morphine as a sedative is bad practice • In principle, there is no artificial administration of fluids during the provision of continuous sedation • Continuous deep sedation differs from euthanasia in that its aim is not to shorten life	

• ^A. The 2009 version of the RDMA guideline was the actual version during the time of the interviews^

## Results

Between September 2019 and December 2020, we interviewed 41 HCPs. Characteristics of the HCPs are listed in Table 2. The interviews lasted between 30 and 93 min, with a mean duration of 59 min. The time between the most recent case of CDS of the HCPs and the interview varied from the same day to months, and was in one case more than a year.

**Table 2:** Respondents’ characteristics

	Number N = 41
**Gender**	
Female	27
Male	14
**Age**	
21–29	1
30–39	4
40–49	11
50–59	17
60–69	8
**Religion**	
Religious	17
Not religious	22
Unknown	2
**Professional background**	
General practitioner	10
Geriatrician	9
Medical specialist^A^	9
Nurse	9
Nurse physician	2
Social worker	1
Medical doctor without further medical training	1
**Place of work (more options possible)**	
Community care	18
Hospice	10
Nursing home	13
Hospital	13
**Work experience as HCP**	
0–9 years	4
10–19 years	7
20–29 years	18
≥ 30 years	10
Unknown	2
**Followed additional training in palliative care**	
Yes^B^	32
No	9
**Number of patients to whom respondent has provided CDS in the last 12 months**	
0	1
1–10	22
11–20	11
> 20	6
Unknown	1

^A: 6 oncologists, 2 pulmonologists, 1 intensivist^

^B: The additional training in palliative care varied from a course of several days to a training of multiple years^

During the coding of the data we identified three key themes: 1) the course and performance of CDS in clinical practice.2) indications to start CDS, and 3) the decision-making process.

### The course and performance of CDS in clinical practice

Nearly all HCPs stated that they were familiar with the RDMA guideline on CDS and stated that they used the guideline as a reference when providing CDS. Midazolam was the medication mostly used as a sedative, administered by repeated injections or by continuous infusions.

HCPs stated that it is not always evident how the symptoms of the patient will evolve over time. Some stated that over time they increasingly used intermittent sedation, a so-called time-out, sometimes resulting in CDS. These HCPs experienced that they did not always have sufficient knowledge of the background of patients, for example during evening and night shifts. The reason to start with intermittent sedation for these HCPs was to relieve time pressure and to create space to evaluate the patient’s symptoms.

*General practitioner: “What hopefully increasingly will be used is intermittent sedation, when there is chaos and pressure, which increases the suffering of the patient. I think it can be a good solution to choose for a single dose in these situations.”*

HCPs mentioned several factors they experienced as supportive in the decision-making and performance of CDS. Factors mentioned were the possibility to discuss options for supportive care and the need to start CDS with a colleague, recurrent team meetings where the use of palliative care and CDS could be discussed, increased experience and knowledge concerning palliative care and CDS, and the RDMA guideline that provides guidance in the decision-making and performance on CDS.

Some HCPs experienced that the use of CDS not always successfully relieved the suffering of a dying patient despite the fact that they increased the dosage of the sedative according the guideline.

*Nurse: “And my last consult, there was a general practitioner who started sedation which did not succeed, it was a young man, who during sedation got up constantly and screamed for help and that he was going to die. There were young kids walking around the bed. Well, some sedations just don’t succeed.”*

### Indications to start CDS

Reporting on their most recent case, the majority of HCPs stated that the indication to start CDS was an accumulation of multiple symptoms leading to a refractory state.

*Nursing home physician: “it was a combination of different factors. There was not just one single symptom, so that you could say, we increase the doses of pain killers. It was not only the pain, it was the total despondency of not getting better anymore. The patient said, I am exhausted, turning in bed already costs me so much energy. I don’t want this anymore, I can’t take this anymore. So it was a combination of pain, which is a physical symptom, exhaustion, and existential suffering.”*

Common physical symptoms mentioned were pain, dyspnoea, restlessness, delirium, fatigue, and nausea. Many HCPs stated that non-physical symptoms also played a role, including fear of dying, difficulties with accepting death and loss of dignity. Especially HCPs working at patients’ homes, stated that over the years their interpretation of refractory suffering had broadened, and that non-physical symptoms more often play a role in the decision-making. For medical specialists working in hospitals, this extension of indications was less evident.

Many HCPs stated that their knowledge and experience with providing CDS increased over the years. Some stated that they use CDS more often because they recognize refractory symptoms better.

*General practitioner: “In the past, when my knowledge was not sufficient enough, I remember that I was muddling along. I remember a case of a man with a delirium with motorically restlessness, and where I realized too late: what could I do? Haloperidol is working, but not on these symptoms. And very late I realized that I just needed to add a benzodiazepine. So, looking back on this case, which is more than six years ago, I let him crawl in his bed too long.”*

Others stated that they use CDS less often because they had experienced that CDS cannot successfully relieve suffering at the end of life in all cases.

### The decision-making process

The imminent death of a patient is often discussed by HCPs with patients and their relatives in advance care planning (ACP) conversations. HCPs in our study differed in their opinion on whether CDS should routinely be discussed in these ACP conversations. Some stated that they do not always discuss CDS with patients and their relatives, certainly not when it is not a relevant option yet. Others stated that they routinely discuss the option of CDS with their patients and their relatives. The HCPs who stated that they routinely discuss the option of CDS in advance with patients, did not experience that due to such conversations they were more inclined to start CDS. These HCPs emphasized the importance of framing the decision to start CDS according the RDMA guideline, namely as a medical decision where medical criteria need to be met.

*General practitioner: “What occasionally happens, is that people have certain expectations of CDS. That people say that they have discussed it with their general practitioner and that they don’t choose euthanasia, but sedation instead. I then explain that it doesn’t work that way, that CDS is not something you can choose, that it is something I decide about when I am their attending physician during the dying process, when I think that it is not possible to provide comfort by other palliative treatment options, and that it is not life-shortening. By giving more information I try to manage their expectations.”*

While most HCPs stated that they consider the decision to start CDS a medical decision, they also emphasized that it is important to involve patients and relatives in the decision-making. The extent to which patients and relatives are involved varied, from taking the initiative to start CDS to providing consent for starting CDS.

*Paramedic : “Eventually the patient said that he couldn’t bear it anymore. This is it, he said. The general practitioner visited the patient on a daily basis, so he just waited for the patient to be at this point. We knew that this patient would die soon. So at the moment that the patient said that he couldn’t bear the pain anymore, and was also disorientated at times as he was also suffering from a terminal delirium, he was well able to indicate that he had reached his limit.”*

A few HCPs stated that they had experienced a situation in which the patient or the relatives asked to start CDS while the respondent was convinced that CDS was not an option (yet), based on the criteria of the RDMA guideline.

*Nursing home physician: “Once I made the mistake that I admitted a patient who had already had a conversation about euthanasia and CDS with his general practitioner. I thought, well, this is good advance care planning of the general practitioner. The patient already received palliative care, but there was absolutely no indication for CDS yet. I gave the patient a leaflet about CDS, so that if there were questions we could discuss these. Whereupon 2 days later his wife came to me and asked: when will you start?”*

Most HCPs in our study felt that over the years suffering at the end of life is less tolerated by patients, their relatives, and sometimes also by other HCPs. Most HCPs experienced that they received more requests to relieve the suffering of dying patients using CDS, and a greater need for information among patients and relatives. This was sometimes experienced as pressure. Influence of the media, where dying is sometimes portrayed as a painless and almost beautiful event, was seen as contributing to the diminished tolerance of suffering.

A large minority of respondents in our study mentioned the following quote from relatives of dying patients:

*“you wouldn’t even let a dog suffer like this would you?”.*

HCPs mentioned that the involvement of many different HCPs in the care of a patient makes it difficult to manage expectations at the end of life. Pressuring factors in the decision-making reported by general practitioners occurred during evening- and nightshifts, when they also attend patients they do not know: lack of time, limited knowledge of the situation of the patient, and limited possibility to consult an expert were mentioned as causing overall pressure.

*General practitioner: “At night there isn’t anyone to consult. There is no palliative care consultant you can call, there is no general practitioner specialized in palliative care you can call, there is no colleague available, and the family is pressuring you to start CDS.”*

Furthermore, most HCPs in our study stated that for patients and relatives differences between euthanasia and CDS are often unclear. HCPs experienced that they need to explain more often what the differences between CDS and euthanasia are, and in which situations CDS and euthanasia can be used. In some cases, euthanasia had been discussed in an earlier phase, but was no longer considered an option by the HCP, because the situation of the patient declined too rapidly. In these cases HCPs also experienced pressure to start CDS.

*Nurse: “He constantly mixed it (euthanasia and CDS) up, and said: I don’t care how you name it, as long as I get my injection and I don’t wake up tomorrow.”*

## Discussion

The aim of our study was to explore potential causes of the rise in the use of CDS in the Netherlands according to HCPs who have been participating in this practice. HCPs in our study mentioned several factors that could have led to a lower threshold to start CDS. The indication to start CDS is often a combination of symptoms resulting in a refractory state [17]. HCPs in our study stated that with growing experience, they had learned to better recognize a refractory state of severe suffering in terminally ill patients.

In addition, they stated that they had started to interpret the concept of refractory state more broadly and more often included symptoms of non-physical origin. Most HCPs experienced more requests to start CDS by patients, their relatives, and sometimes by other HCPs involved, and felt that over the years suffering at the end of life is less tolerated by patients, their relatives, and sometimes also by other HCPs. Some HCPs in our study experienced more pressure from patients and relatives to start CDS. HCPs also stated that for patients and their relatives differences between euthanasia and CDS may be unclear.

The RDMA guideline describes CDS as an intervention that is based on a medical decision where medical criteria need to be met [12]. The broader interpretation of refractory suffering makes it more difficult to interpret the decision to start CDS as solely a medical decision. Studies show that HCPs in other countries also seem to have embraced a broader interpretation of indications for sedation [13, 14]. There seems to be a greater acceptance for suffering of non-physical origin as a ground for starting CDS [14].

HCPs in our study mentioned several reasons for a decreased tolerance for suffering among patients, their relatives and HCPs at the end of life. First, they mentioned the role of the media. HCPs stated that dying in the media is sometimes portrayed as a painless and beautiful event, which has also been shown in previous studies [18, 19]. Other studies proved that a substantial proportion of patients experience symptoms at the end of life, including pain, shortness of breath and fatigue [20, 21]. It could be that due to the media, patients and their relatives incorrectly expect that they will not experience symptoms at the end of life, and when they do face such symptoms, they more often request CDS.

Second, HCPs in our study mentioned that differences between CDS and euthanasia are not always evident for patients and relatives. Since 2002 it has been established by Dutch law, that HCPs may provide euthanasia for patients under strict conditions [22, 23]. There needs to be a well-considered and voluntary request of the patient, there must be unbearable suffering without any prospect of relief, and an independent physician must assesses the patient’s request [22, 23]. It could be that an increased awareness of the option of euthanasia, also increased the awareness for other options to relieve suffering at the end of life, including CDS.

Third, some HCPS stated that over the years they had discussed the option of CDS more often in ACP conversations with patients and their relatives. Little is known about the impact of these conversations on patients and their families’ expectations concerning CDS. The HCPs in our study who discussed CDS in these conversations did not experience an increased number of requests for CDS. However, when HCPs discuss patient wishes regarding CDS in an earlier stage, an expectation may be created that CDS can indeed be started upon request in case of suffering.

Fourth, some HCPs stated that they increasingly used intermittent sedation to relieve suffering. The use of intermittent sedation to relieve suffering of terminally ill patients is reported in several studies, but little is known about the transition from intermittent to continuous sedation when the use of intermittent sedation is not effective [24, 25]. It could be that the use of intermittent sedation more often leads to the use of CDS when the first is not sufficiently effective.

### Strengths and limitations of this study

This qualitative study is one of the few studies that provides insight in the experiences and practices of HCPs with providing CDS. The diversity of HCPs from different settings is a strength of our study. The majority of the respondents had multiple years of experience with providing CDS and were able to reflect on their evolving practices and experiences. By systematically asking details about the most recent case, we tried to get a more general insight in their practice than when we would have discussed the most memorable case. The clarity about the definition of CDS we provided at the start of the interview can also be considered a strength.

A limitation of our study is potential selection bias. Most respondents had had additional training in palliative care, worked on a daily basis with terminally ill patients, and had a special interest in the topic. They were mainly nurses and physicians. Spiritual carers were also invited, but did not participate. Another limitation of our study is the risk of recall bias. In our study, we asked the respondents to describe their most recent case of CDS, which was for some of the respondents several months ago. Lastly, we describe practices and experiences of the use of CDS from only the HCP perspective and not from the perspective of relatives of patients who received CDS.

### Conclusions and implications

This study provides insight into how participating HCPs perceive that their practice of CDS changed over time. The combination of a broader interpretation of refractory suffering by HCPs and a decreased tolerance of suffering at the end of life by patients, their relatives and HCPs, may have led to a lower threshold to start CDS. Results of our study underpin the importance of discussing the option of CDS in conversations between HCPs, patients and relatives. In future research, it would be valuable to explore patients’ and relatives’ experiences and expectations on the use of CDS.

### Electronic supplementary material

Below is the link to the electronic supplementary material.

Supplementary Material 1

## Data Availability

The datasets generated and analysed during current study are not publicly available with respect to the privacy of the HCPs, but are available from the corresponding author upon reasonable request.

## Abbreviations

CDS: continuous deep sedation
HCP: health care provider
