# Supplementary material for: Continuous deep sedation at the end of life: a qualitative interview-study among health care providers on an evolving practice
Source: BMC Palliat Care. 2023 Oct 26;22:160. doi: 10.1186/s12904-023-01289-z (PMC10601190; doi:10.1186/s12904-023-01289-z)
Supplement: Supplementary file 1 — Supplementary Material 1 [file 12904_2023_1289_MOESM1_ESM.pdf]

**Interview guide, semi-structured interviews with health care professionals on the use of continuous deep sedation**	
<u>Definition</u>	
Can you explain what you consider as palliative sedation?<br><br>Definition of palliative sedation: In this interview we want to elaborate on the most far-reaching form of sedation: the use of continuous deep sedation until the end of life (CDS).	
<u>Reflecting on the health care professional's most recent case of CDS</u>	
Health care professional's most recent case of CDS	- Introduction by the health care professional of their most recent case of CDS<br>- Wat was the reason to consider the use of CDS?<br>- How did the decision-making take place?<br>- Did you experience pressure?<br>- What was the estimated life expectancy of the patient?<br>- How did the sedation proceed?<br>- Can you tell something about how the sedation was performed?<br>- How do you look back on the dying process of the patient and the use of CDS?
<u>Changed practices in the use of CDS</u>	
CDS in clinical practice	- Did your practice of how to provide CDS change?<br>- Do you discuss the use of CDS often with your patients?<br>- What is your experience of what patients and their relatives know about the use of CDS?<br>- What are their expectations of CDS?<br>- Did your point of view on the use of CDS change?<br>- For which indications do you mostly provide CDS?<br>- For which patient groups do you usually start CDS? For example<br>- Did the decision-making process change compared to 5 years ago?<br>- Do you use the national guideline on the use of CDS?
<u>Quotes on the opinions and experiences of the health care professionals</u>	
Quotes	- In my opinion, CDS is not much more than a normal part of palliative care<br>- The transition from symptom relief towards CDS is usually a slippery slope<br>- In my opinion, it is important to discuss the use of CDS in conversations with patients on the dying process,<br>- My patients are less able to cope with severe symptoms than 5 years before.<br>- In my opinion patients experience greater need to be in control of their own dying process compared to 5 years before.<br>- I experience a greater need to be in control of the dying process of patients compared to 5 years before.<br>- A lot of my patients consider the use of CDS as a mild form of euthanasia (passive euthanasia or euthanasia light)<br>- Over the years, I've widened my interpretation of refractory suffering<br>- As health care professional I consider the use of CDS as a mild form of euthanasia (passive euthanasia or euthanasia light)<br>- In my opinion, palliative sedation is a medical answer to a medical problem
<u>Finishing the interview</u>	
